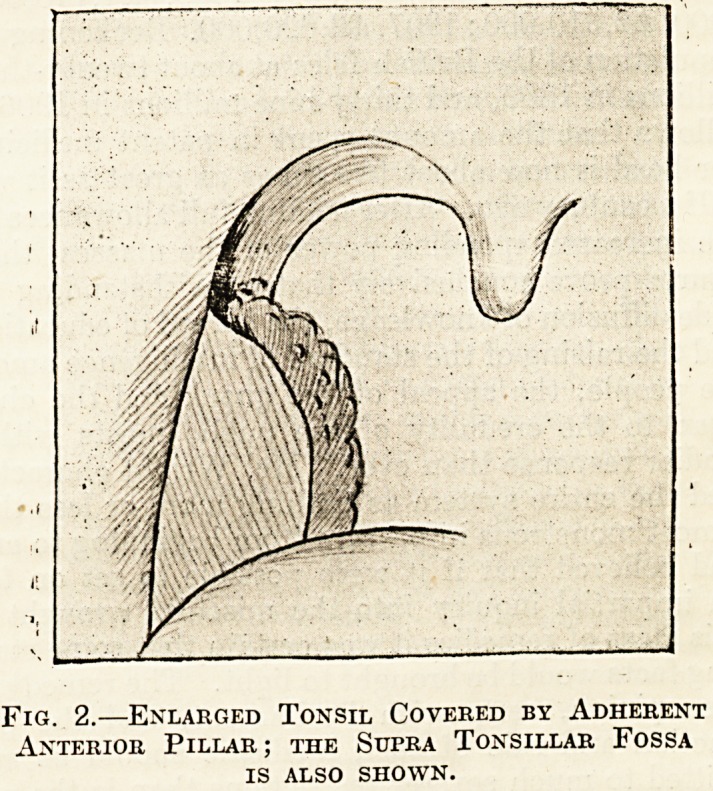# Enlargement of the Tonsils—II

**Published:** 1908-09-19

**Authors:** 


### Laryngology and Rhinology.

### ENLARGEMENT OF THE TONSILS—II.

Before entering on the subject of the operative treatment of chronic tonsillar enlargement, it will be advisable to discuss a few details of the anatomy of these bodies which are of practical application. The tonsils, then, are a pair of almond-shaped bodies placed each in an elongated cleft between the anterior and posterior pillar of the fauces. Externally, the tonsil corresponds roughly in position with the angle of the jaw; its external surface lies upon the floor of the cleft, which is formed by the pharyngeal

Fig. 1.—External Surface of Tonsil after Removal, showing the Cut Surface.

aponeurosis, and from which it may be peeled as from a capsule; this aponeurosis resists the extension of a tonsillar malignant growth into the tissues of the neck. Outside this is the superior constrictor of the pharynx, and, external again to this, a space filled with loose areolar tissue separates it from the internal pterygoid muscle and the angle of the jaw. The great vessels pass upwards in this space, but at a very considerable distance external to and behind the tonsil; fears of wounding the internal carotid artery while removing the tonsil may be considered as quite unfounded, for the vessel lies quite an inch from the base of the gland. The ascending pharyngeal and the facial arteries are both nearer, but it is nevertheless probable that the severe bleeding which occasionally occurs proceeds from the branches which supply the tonsil.

Below, the tonsil fills the cleft between the two faucial pillars and extends well below the level of the dorsum linguæ, even to the glosso-epiglottic folds, so that to remove the lower part the guillotine must be directed distinctly downwards. When the tonsil is enlarged it generally grows downwards below the edge of the cleft, and so becomes pedunculated: thus, when excised with the guillotine, it is seen to have a cut surface considerably smaller than its greatest bulk and placed eccentrically near the upper end; when this is found to be the case it may be assumed that the tonsil has been satisfactorily removed.

In contradistinction, the upper extremity of the tonsil does not reach into the end of the cleft; a triangular hollow, called the "supratonsillar fossa," is thus formed. It is partly covered in by the expanded anterior pillar of the fauces, and it sometimes extends downwards for a considerable distance between the tonsil and the pharyngeal aponeurosis, and plays an important part in the pathology of quinsy. Several of the tonsillar crypts open into it, and it may become filled with their decomposing secretion. When the tonsil is diseased it frequently becomes adherent to the anterior pillar of the fauces, which is spread out over its surface. A tonsil thus "buried" is somewhat difficult to remove.

In discussing the operative treatment we must not be taken to imply that every enlarged tonsil requires to be removed. The indications may be gathered from the account of the symptoms in the preceding article. Direct indications are recurring attacks of inflammation, signs of septic absorption or cervical adenitis, while enlarged tonsils co-existing with adenoids should in general be removed at the same time. Tonsils should not be removed while acutely inflamed, though this procedure may be called for if they become so large as to interfere with breathing or with swallowing. It is true that healing generally occurs readily under such circumstances, but it is nevertheless safer to wait. In the case of flat tonsils which only swell up when inflamed, their removal

Fig. 2.—Enlarged Tonsil Covered by Adherent Anterior Pillar; the Supra Tonsillar Fossa is also shown.

has been advised as easier in this condition; but for such cases methods of removal other than by use of the guillotine are to be advised. In cases of chronic follicular tonsillitis the crypts may be slit up and emptied one by one; but if many crypts are affected it is far better to remove the tonsil. The galvano-cautery must be mentioned as affording an alternative method of treatment; a broad electrode should be used and three or four punctures made at each sitting under local anæsthesia. The method is very tedious, as eight or ten sittings are often necessary at intervals of four to seven days, and considerable soreness results; it is only to be advised in cases of hæmophilia, in some flat fibrous tonsils in adults where hæmorrhage may be feared, and for patients who refuse any cutting operation.

*(To be continued.)*

## Figures and Tables

**Fig. 1 f1:**
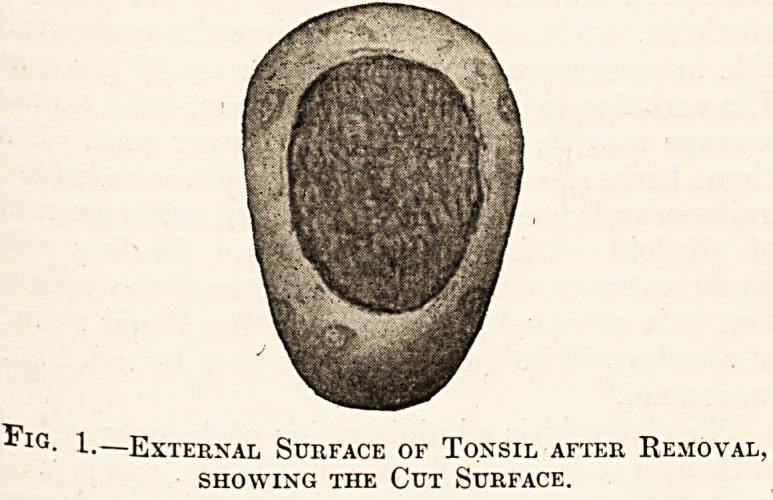


**Fig. 2 f2:**